# Supplementary material for: Pathogen-derived extracellular vesicles mediate virulence in the fatal human pathogen Cryptococcus gattii
Source: Nat Commun. 2018 Apr 19;9:1556. doi: 10.1038/s41467-018-03991-6 (PMC5908794; doi:10.1038/s41467-018-03991-6)
Supplement: Supplementary file 1 — Supplementary Information [file 41467_2018_3991_MOESM1_ESM.pdf]

Supplementary Information

Pathogen-derived extracellular vesicles mediate virulence in the fatal human pathogen *Cryptococcus gattii*

Bielska *et* al.

Supplementary Figure 1

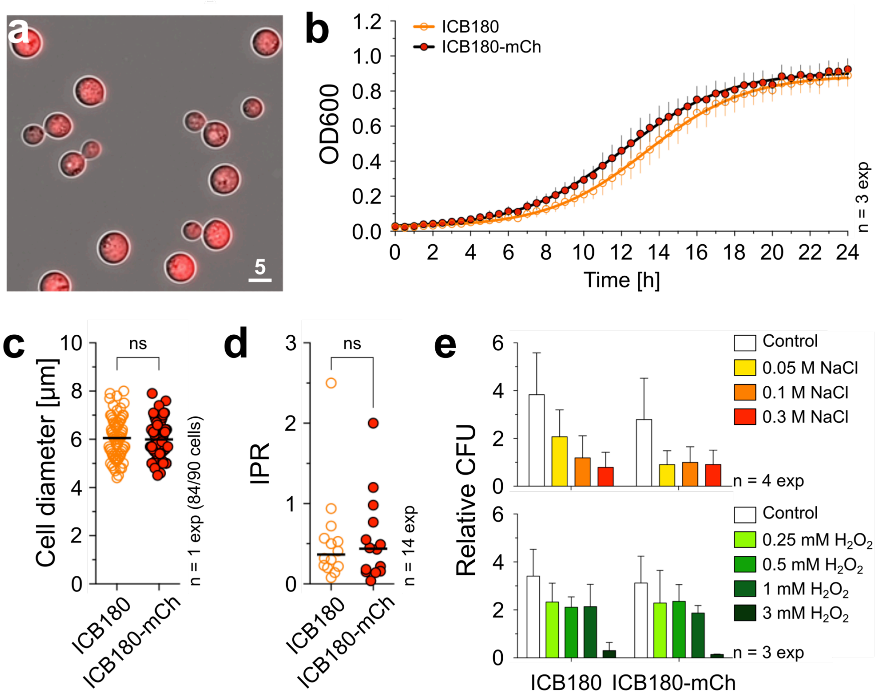

Supplementary Figure 1: Constitutive expression of a red fluorescent protein, mCherry, in ICB180 does not impair cell morphology, growth or survival under stress conditions

a, Epifluorescence image showing expression of a red fluorescent protein mCherry by ICB180 cells in a liquid YPD culture. Bar: 5 µm.

b, Growth curves of ICB180 and ICB180-mCherry strains in liquid YPD medium at 37°C. Constitutive mCherry expression has no influence on growth ($P = 0.1799$, linear regressions calculated for values obtained during exponential phase of growth, from 7 to 18 hours). Data are presented as means ± s.d. and are representative of results from 3 independent experiments.

c, Cell size is not changed after expression of a red fluorescent protein by ICB180 (5.99 ± 0.66 µm in diameter) when compared to the wild type strain ICB180 (6.06 ± 0.81 µm in diameter). *ns* indicates statistically non-significant difference ($P = 0.5720$, unpaired Student's t-test with Welch's correction). Data are presented as scattered dot plots with lines representing their medians and are representative of results from 84-90 cells.

d, Survival of ICB180 strain constitutively expressing mCherry does not affect its intracellular proliferation rate (IPR) after macrophage infection. *ns* indicates statistically non-significant difference ($P = 0.8528$, Wilcoxon matched-pairs signed rank test). Data are presented as scattered dot plots with lines representing their medians. Data are

representative of results from 14 independent experiments with 412-1003 total number of yeasts counted for each sample.

e, Relative colony forming units (CFU) values were calculated from CFUs obtained at time point 0 and 24 hours growing in the presence of different concentrations NaCl or H_2O_2. Data are presented as mean ± s.d. and are representative of results from 4 (for NaCl) and 3 (for H_2O_2) independent experiments.

Supplementary Figure 2

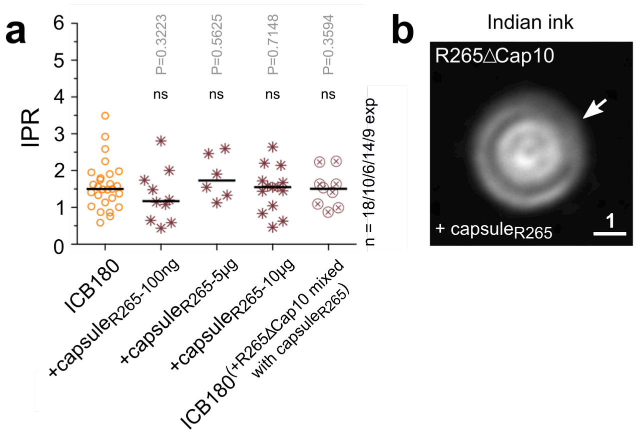

Supplementary Figure 2: Capsular material obtained in a method without an autoclaving step is not sufficient to increase the IPR of non-outbreak cryptococci

a, IPR of a non-virulent ICB180 strain is not altered by the presence of capsular material isolated from R265 (+capsule$_{R265\text{-}100\ ng}$; +capsule$_{R265\text{-}5\ \mu g}$; +capsule$_{R265\text{-}10\ \mu g}$) lacking the autoclaving step. Lack of increase of the IPR was also observed after *in vitro* recovery of the capsule by the acapsular cells in the transwell system (ICB180$^{(+R265\Delta Cap10\ mixed\ with\ capsuleR265)}$; see also Supplementary Figure 2b). Data are presented as scattered dot plots with lines representing their medians. Individual Wilcoxon matched-pairs signed rank test presented as P values above each dot plot. Note lack of significant differences between the samples where *ns* (P > 0.05), not significantly different. Data are representative of results from at least 6 independent experiments with 828-3783 total number of yeasts counted for each sample.

b, India Ink image of acapsular R265ΔCap10 mixed for 1 hour with capsule isolated from 10-days culture of R265 without the autoclaving step. Note the presence of recovered polysaccharide capsule (arrow). Bar: 1 µm.

Supplementary Figure 3

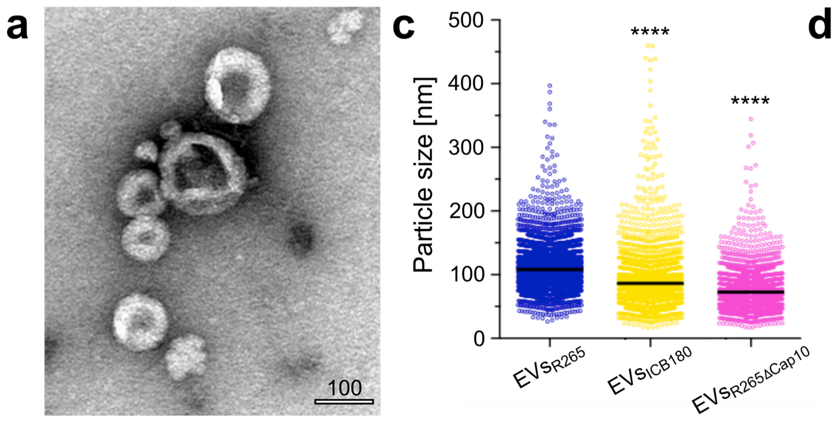

d

Particle size	EVsR265	EVsICB180	EVsR265Δ Cap10
Min size [nm]	26.28	16.97	17.08
<25 nm	0.00%	0.51%	0.90%
25-49 nm	1.80%	10.63%	17.63%
50-74 nm	10.25%	24.30%	33.65%
75-99 nm	26.06%	31.95%	26.99%
100-124 nm	31.15%	16.04%	13.22%
125-149 nm	16.51%	7.09%	4.21%
>150 nm	14.22%	9.47%	3.41%
Max size [nm]	397.1	838	344.4
**Median size [nm]**	**108.2**	**86.26**	**72.75**

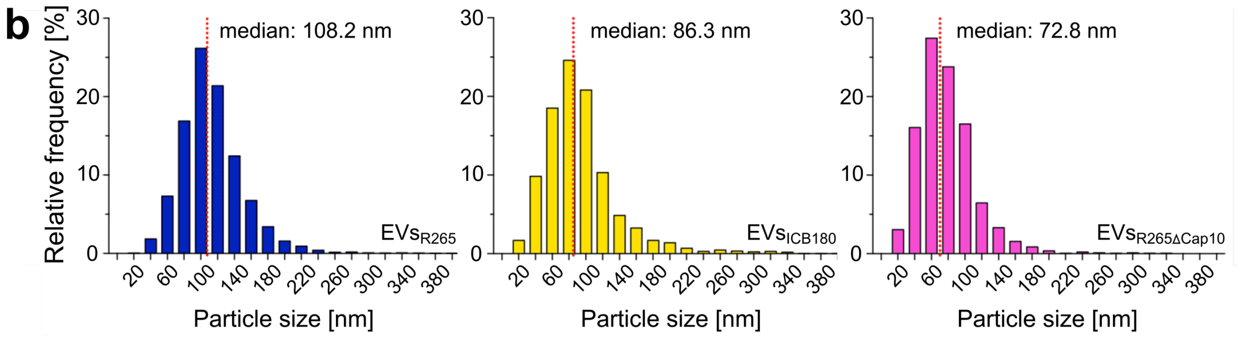

Supplementary Figure 3: *C. gattii* releases extracellular vesicles that differ in size depending on the strain

a, Transmission electron micrograph of cell-derived vesicles isolated by ultracentrifugation from the Pacific Northwest outbreak strain R265. Bar: 100 nm.

b, Histograms showing the range of particle sizes from R265 (blue), ICB180 (yellow) and R265ΔCap10 (purple) measured by NanoSight with red dotted lines representing the median.

c, Scatter plots of particle sizes of EVs isolated from R265 (blue), ICB180 (yellow) and R265ΔCap10 (purple) strains. Horizontal lines represent medians. Mann-Whitney test, **** $P \leq 0.0001$; n=3101 EVs_{R265} (from 9 isolations), 2144 EVs_{ICB180} (from 4 isolations) and 1997 $EVs_{R265\Delta Cap10}$ (from 5 isolations).

d, Table summarizing results obtained from nanoparticle tracking analysis for EVs isolated from R265, ICB180 and R265ΔCap10.

Supplementary Figure 4

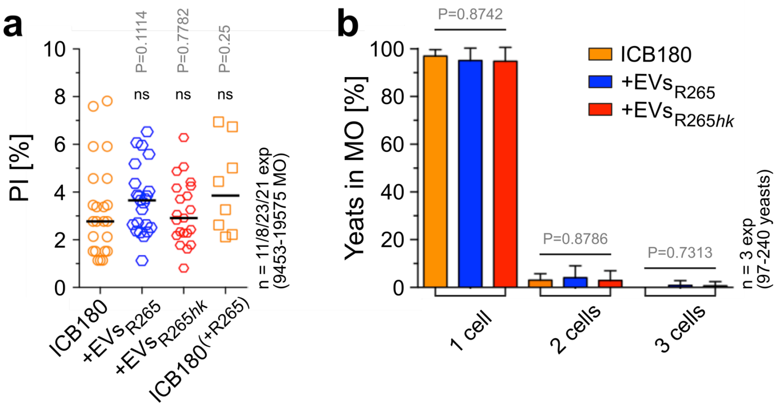

Supplementary Figure 4: The presence of conditioned media or EVs isolated from R265 does not increase the phagocytosis of ICB180 by murine macrophages

a, Phagocytic index (PI) is shown as a percentage of macrophages with intracellular ICB180-mCh within the first two hours post-infection and does not differ significantly between macrophages containing ICB180, ICB180 in the presence of a transwell containing R265 (ICB180$^{(+R265)}$), or ICB180 in the presence of EVs isolated from R265 (+EVs$_{R265}$) or ICB180 in the presence of heat inactivated EVs isolated from R265 (+EVs$_{R265\textit{hk}}$). Data are presented as scatter dot plots with lines representing the median. Data are representative of results from 8-23 independent experiments with a minimum of 226 macrophages analysed per sample per experiment. Wilcoxon paired t-test where *ns* stands for 'non-significant' ($P > 0.05$).

b, Number of ICB180 yeast cells phagocytosed by macrophages in the first two hours post-infection does not differ significantly in the presence or in the absence of EVs isolated from R265 (+EVs$_{R265}$) or heat inactivated EVs isolated from R265 (+EVs$_{R265\textit{hk}}$). Majority of the infected macrophages contain only one yeast cell. All numbers are given as means ± s.d. and are representative of results from 3 independent experiments with at least 3 biological replicates of EVs with a minimum of 226 macrophages analysed per sample per experiment. P values (shown above) were analysed by Kruskal-Wallis tests.

Supplementary Figure 5

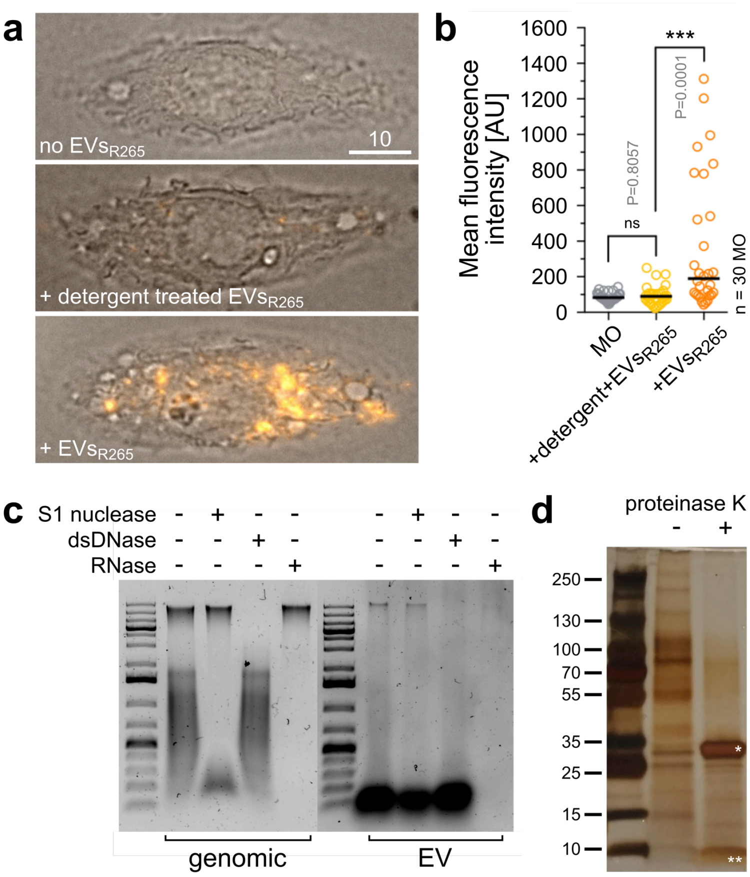

Supplementary Figure 5: Abundance of lipids, nucleic acids and proteins in EVs can be highly reduced using sodium deoxycholate, DNase/RNase or proteinase K

a, Macrophages alone (no EVs_{R265}, top) and macrophages incubated for 30 min with EVs_{R265} prior treated with sodium deoxycholate and later stained with a fluorescent lipophilic dye Vybrant DiI (+ detergent treated EVs_{R265}, middle) do not show typical fluorescent signals as macrophages incubated with EVs_{R265} stained with Vybrant DiI (+ EVs_{R265}, bottom) due to the lack of exosomal lipids after detergent treatment necessary for incorporation of a lipophilic dye. Pictures represent maximum intensity projections of 26 z-stacks obtained from 2.5 µm cross section through macrophages. Bar: 10 µm.

b, Graph showing mean fluorescent intensities from macrophages alone (MO) and from macrophages incubated with R265 capsule ($capsule_{R265}$) or EVs isolated from R265 (EVs_{R265}) without detergent (no Triton X-100) or with detergent treatment (0.1% Triton X-100, 1% Triton X-100). Data are presented as scattered dot plots with lines representing their medians. Data are representative of results from 1 experiment with 30 macrophages analysed per sample. Unpaired Mann-Whitney tests where *** ($P \leq 0.001$), significant difference and *ns* ($P > 0.05$), not significantly different.

c, Nucleic acids isolated from R265 cells (genomic) and from 300 μg of EVs_{R265} (exosomal) using Wizard Genomic DNA Purification Kit (Promega) without added RNase A can be visualized on an agarose gel. Top bands represent DNA, while bottom smears represent RNA. Presence of S1 nuclease highly reduced RNA smears in genomic sample, but only slightly in exosomal sample. Addition of dsDNase removed the top bands of nucleic acids in both samples. RNase cocktail completely removed smear bands from genomic and exosomal samples.
d, 50 μg of control EVs_{R265} and EVs_{R265} treated with proteinase K were loaded on a 4–20% protein gel and silver stained (Pierce Silver Stain). Note a high reduction of protein bands, an additional band of 28.9 kDa belonging to proteinase K (*) and much higher number of peptides in the EVs sample treaded with proteinase K as a result of protein digestion (**).

Supplementary Figure 6

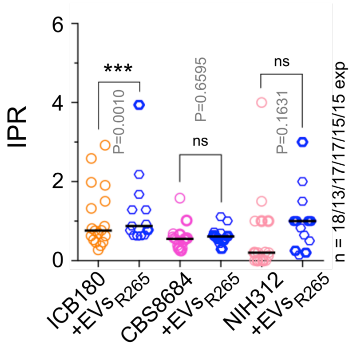

Supplementary Figure 6: EVs isolated from R265 do not enhance proliferation rates of very low in virulence *C. gattii* strains
The presence of 10 μg or 50 μg of EVs (symbols with thicker borders) isolated from R265 does not increase the proliferation of CBS8684 or NIH312 inside murine macrophages. Data are presented as scatter dot plots with lines representing the median. As a control ICB180 strain was used (ICB180, $+EVs_{R265}$). Data are representative of results from at least 13 independent experiments with 51 (for NIH312)-2404 total number of yeasts counted for each sample. Wilcoxon paired t-test where *** ($P \leq 0.001$), significant difference and *ns* ($P > 0.05$), not significantly different.

Supplementary Figure 7

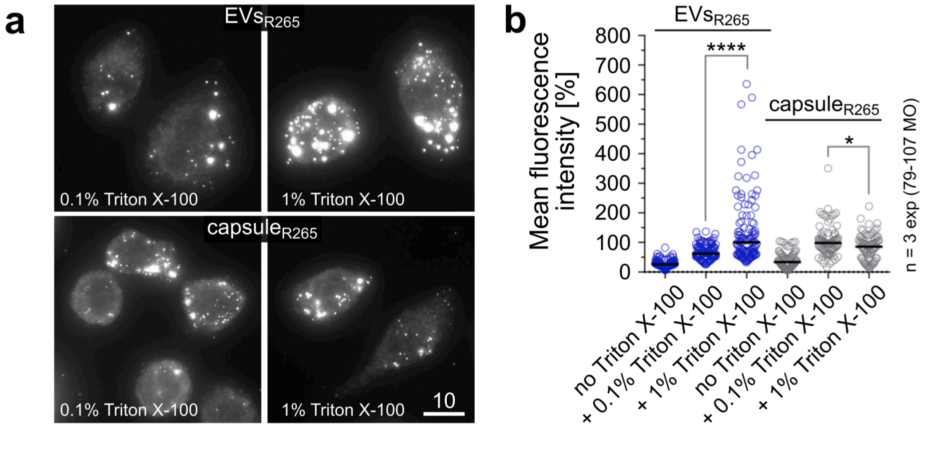

Supplementary Figure 7: Internalised EVs are more resistant to detergent treatment than internalised capsule

a, Increasing Triton X-100 concentration from 0.1 to 1% leads to further permeabilization of internalised EVs and to increased immunofluorescence in macrophages (EVs_{R265}) but not in cells exposed only to purified capsular material ($capsule_{R265}$). Pictures are maximum intensity projections of 21 z-stacks obtained from 10 µm cross section through macrophages. Bar: 10 µm.

b, Graph showing normalized percentages of mean fluorescent intensities from macrophages incubated with R265 capsule ($capsule_{R265}$) or EVs isolated from R265 (EVs_{R265}) without detergent (no Triton X-100) or with detergent treatment (0.1% Triton X-100; 1% Triton X-100). Data are presented as scattered dot plots with lines representing their medians. Data are representative of results from 3 independent experiments with a minimum of 25 macrophages analysed per sample per experiment. Unpaired Mann-Whitney tests where * ($P = 0.0155$), significant difference and **** ($P \leq 0.0001$), highly significant difference.

Supplementary Figure 8

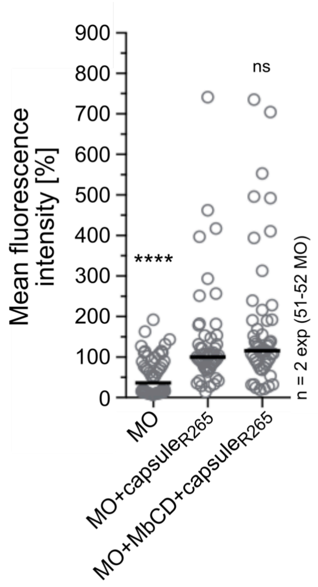

Supplementary Figure 8: The uptake of cryptococcal capsule by J774 macrophages is not inhibited by using a raft-specific inhibitor methyl-β-cyclodextrin ($MO+MbCD+capsule_{R265}$)

The graph shows normalized percentages of mean fluorescent intensities from macrophages alone (MO) or incubated with MAb 18B7-immunostained capsule isolated from R265 ($MO+capsule_{R265}$). The fluorescence intensities were not significantly different when macrophages were treated with methyl-β-cyclodextrin prior the incubation with cryptococcal capsule. Data are presented as scattered dot plots with lines representing their medians. Data are representative of results from 2 independent experiments with a minimum of 25 macrophages analysed per sample per experiment. Unpaired Mann-Whitney tests where **** ($P \leq 0.0001$), highly significant difference and *ns* ($P = 0.1318$), not significantly different.

Supplementary Table 1: Strains and plasmids used in the study

<table>
<tr><th>Strain/Plasmid</th><th>Sero -type</th><th>Geno -type</th><th>AFLP</th><th>Description</th><th>Source</th></tr>
<tr><td>C. gattii R265</td><td>B</td><td>VGIIa</td><td>6</td><td>Clinical isolate from Duncan, British Columbia, Canada</td><td>[1]</td></tr>
<tr><td rowspan="2">C. gattii R265-GFP</td><td>B</td><td>VGIIa</td><td>6</td><td rowspan="2">GFP expressing R265 (R265_GFP6)</td><td rowspan="2">[2]</td></tr>
<tr><td colspan="3">R265 / Pact1-egfp, hyg^R</td></tr>
<tr><td>C. gattii ICB180</td><td>B</td><td>VGII</td><td>6</td><td>Environmental isolate (eucalyptus tree) from Sao Paulo, Brazil</td><td>[3]</td></tr>
<tr><td>C. neoformans KN99α</td><td>A</td><td>VNI</td><td>1</td><td>Congenic strain with the α strain H99 (clinical isolate, USA)</td><td>[4]</td></tr>
<tr><td>C. gattii CBS8684</td><td>B</td><td>VGII</td><td>6</td><td>Environmental isolate (communal wasp) from Quebrada de los Cuervos, Uruguay</td><td>[5,6]</td></tr>
<tr><td>C. gattii NIH312</td><td>C</td><td>VGIII</td><td>5</td><td>Human cerebrospinal fluid</td><td>[7]</td></tr>
<tr><td rowspan="2">C. gattii ICB180-mCherry</td><td>B</td><td>VGII</td><td>6</td><td rowspan="2">mCherry expressing ICB180</td><td rowspan="2">This study</td></tr>
<tr><td colspan="3">ICB180 / Phis3-mcherry, hyg^R</td></tr>
<tr><td>C. gattii R265ΔCap10</td><td>B</td><td>VGIIa</td><td>6</td><td>Acapsular mutant of R265</td><td>[8]</td></tr>
<tr><td>C. gattii R265ΔCap10-GFP</td><td>B</td><td>VGIIa</td><td>6</td><td>GFP expressing R265ΔCap10</td><td>This study</td></tr>
<tr><td>pAG32_GFP</td><td colspan="3">Pact1-egfp, hyg^R</td><td>GFP plasmid</td><td>[2]</td></tr>
<tr><td>pRS426H-CnmCherry</td><td colspan="3">Phis3-mcherry, hyg^R</td><td>mCherry plasmid</td><td>[9]</td></tr>
<tr><td colspan="6">P, promoter; /, ectopically integrated; hyg^R, hygromycin resistance; act1, actin1; his3, histone 3; GFP, green fluorescent protein; AFLP, amplified fragment length polymorphism</td></tr>
</table>

Supplementary Information References

1 Kidd, S. E. *et al.* A rare genotype of *Cryptococcus gattii* caused the cryptococcosis outbreak on Vancouver Island (British Columbia, Canada). *Proceedings of the National Academy of Sciences of the United States of America* **101**, 17258-17263, doi:10.1073/pnas.0402981101 (2004).

2 Voelz, K., Johnston, S. A., Rutherford, J. C. & May, R. C. Automated analysis of cryptococcal macrophage parasitism using GFP-tagged cryptococci. *PLoS One* **5**, e15968, doi:10.1371/journal.pone.0015968 (2010).

3 Barreto de Oliveira, M. T. *et al. Cryptococcus neoformans* shows a remarkable genotypic diversity in Brazil. *Journal of clinical microbiology* **42**, 1356-1359 (2004).

4 Nielsen, K. *et al.* Sexual cycle of *Cryptococcus neoformans var. grubii* and virulence of congenic a and alpha isolates. *Infection and immunity* **71**, 4831-4841 (2003).

5 Hagen, F. *et al.* Ancient dispersal of the human fungal pathogen *Cryptococcus gattii* from the Amazon rainforest. *PloS one* **8**, e71148, doi:10.1371/journal.pone.0071148 (2013).

6 Fraser, J. A. *et al.* Same-sex mating and the origin of the Vancouver Island *Cryptococcus gattii* outbreak. *Nature* **437**, 1360-1364, doi:10.1038/nature04220 (2005).

7 Fraser, J. A., Subaran, R. L., Nichols, C. B. & Heitman, J. Recapitulation of the sexual cycle of the primary fungal pathogen *Cryptococcus neoformans var. gattii*: implications for an outbreak on Vancouver Island, Canada. *Eukaryotic cell* **2**, 1036-1045 (2003).

8 Hu, G. & Kronstad, J. W. Gene disruption in *Cryptococcus neoformans* and *Cryptococcus gattii* by *in vitro* transposition. *Current genetics* **49**, 341-350, doi:10.1007/s00294-005-0054-x (2006).

9 Gibson, R. H. *et al.* Mycophenolate mofetil increases susceptibility to opportunistic fungal infection independent of lymphocytes. *bioRxiv*, doi:10.1101/131540 (2017).
