# Supplementary material for: Pathogen-derived extracellular vesicles mediate virulence in the fatal human pathogen Cryptococcus gattii
Source: Nat Commun. 2018 Apr 19;9:1556. doi: 10.1038/s41467-018-03991-6 (PMC5908794; doi:10.1038/s41467-018-03991-6)
Supplement: Supplementary file 3 — Description of Additional Supplementary Files [file 41467_2018_3991_MOESM3_ESM.pdf]

Description of Additional Supplementary Files

File Name: Supplementary Movie 1
Description: Three-dimensional visualisation of fungal EVs_{R265} immunostained in MAb 18B7 in a murine macrophage after 30-minutes incubation. Bar: 5 µm.
